# Supplementary material for: uPAR (PLAUR) Marks Two Intra-Tumoral Subtypes of Glioblastoma: Insights from Single-Cell RNA Sequencing
Source: Int J Mol Sci. 2024 Feb 7;25(4):1998. doi: 10.3390/ijms25041998 (PMC10889167; doi:10.3390/ijms25041998)
Supplement: Supplementary file 1 [file ijms-25-01998-s001.zip › Supplementary_figure.pdf]

Supplementary Material

1 SUPPLEMENTARY FIGURE

1.1 Figures

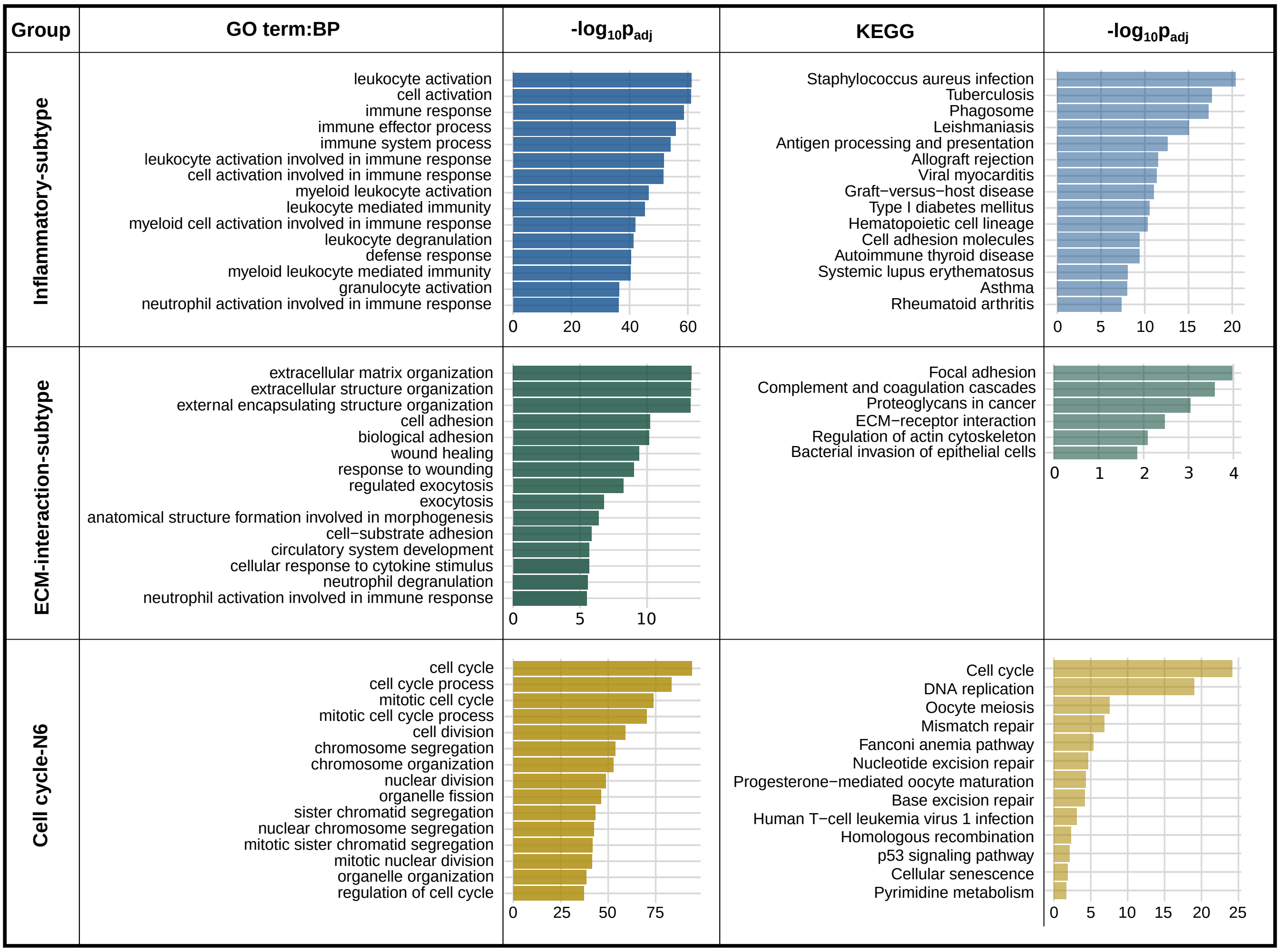

Figure S1. The enrichment results including N6.
